# Safety profile of herpes zoster vaccines in the United States: a descriptive and disproportionality analysis of adverse events reported to the vaccine adverse event reporting system (VAERS), 2017–2024

**DOI:** 10.3389/fmed.2026.1920596

**Published:** 2026-08-13

**Authors:** Mohammed Alkharaiji, Salahaden R. Sultan, Yousif A. Kariri, Saleh Alzughaibi, Ahmed Hazazi, Mohammed A. Alsuliman, Mansour Alsaleem, Thamir M. Alshammari

**Affiliations:** 1 Department of Public Health, College of Health Sciences, Saudi Electronic University, Riyadh, Saudi Arabia; 2 Department of Radiologic Sciences, Faculty of Applied Medical Sciences, King Abdulaziz University, Jeddah, Saudi Arabia; 3 Department of Clinical Laboratory Sciences, College of Applied Medical Sciences, Shaqra University, Shaqra, Saudi Arabia; 4 Department of Health Informatics, College of Health Sciences, Saudi Electronic University, Riyadh, Saudi Arabia; 5 Unit of Scientific Research, Applied College, Qassim University, Buraydah, Qassim, Saudi Arabia; 6 Department of Pharmacy Practice, College of Pharmacy, Jazan University, Jazan, Saudi Arabia; 7 Pharmacy Practice Research Unit, College of Pharmacy, Jazan University, Jazan, Saudi Arabia

**Keywords:** herpes zoster vaccine, pharmacovigilance, shingles, shingrix, vaccine safety, VAERS, zostavax

## Abstract

**Background:**

Herpes zoster causes substantial morbidity in older adults, and US herpes zoster vaccination shifted from the live-attenuated Zostavax to the recombinant Shingrix after 2017 We describe the post-marketing adverse-event profile of these vaccines reported to the US Vaccine Adverse Event Reporting System (VAERS) and identify events reported disproportionately for herpes zoster vaccines.

**Methods:**

In a descriptive cross-sectional design, we analyzed 69,822 US VAERS reports listing a herpes zoster vaccine, received 1 January 2017–25 April 2024. Reports were characterized by demographics, brand, MedDRA preferred terms and system organ classes, and US FDA serious-event indicators; brand subgroups were summarized descriptively. Reporting odds ratios (RORs) with 95% confidence intervals compared the combined herpes zoster vaccine cohort with all other US VAERS reports. Reporting followed the STROBE guideline.

**Results:**

Shingrix accounted for 58,813 (84.2%) reports and Zostavax for 10,507 (15.0%). Reports were predominantly in women (63.9%) and adults aged 50–69 years (median age 64.0 years). Reactogenic and injection-site terms predominated—pyrexia (18.3%), pain (17.8%), chills (15.9%), headache (15.4%), and injection-site pain (14.2%). Against all other US reports, injection-site and influenza-like reactions were modestly enriched (e.g., injection-site pain ROR 2.92), whereas vesicular rash was strongly over-reported (ROR 43.70); the latter was concentrated in live-vaccine reports and reflects reported vaccine-strain disease rather than an unexpected adverse reaction. Serious reports comprised 6.6% of the cohort (2.9% Shingrix, 27.6% Zostavax).

**Conclusion:**

Eight years of VAERS data are consistent with the recognized reactogenicity of the recombinant vaccine and the live-vaccine profile of Zostavax; most reported events were transient and non-serious. These hypothesis-generating disproportionality signals require confirmation in active surveillance with denominator data.

## 1 Introduction

Herpes zoster (HZ), commonly called shingles, is a painful, typically unilateral vesicular eruption that arises when latent varicella-zoster virus reactivates from sensory ganglia where it has resided since primary infection (1). Approximately one in three adults in the United States will develop shingles during their lifetime, with an estimated one million incident cases occurring annually (2). Incidence rises steeply after age 50 and is highest among adults in their seventies and eighties, a pattern that reflects age-related decline in varicella-zoster virus–specific cell-mediated immunity, as well as immunosuppression from coexisting illness or therapy (3, 4). The disease imposes a substantial public-health and economic burden in the United States: a national synthesis estimated that the aggregate annual cost of HZ exceeds US $2.4 billion when direct medical care, productivity losses, and the disability associated with chronic pain are accounted for (5).

The most common and most disabling complication of shingles is postherpetic neuralgia, a chronic neuropathic pain syndrome that develops in 5% to 30% of patients and may persist for months or years after the rash has resolved (6). Other important complications include herpes zoster ophthalmicus with the potential for vision loss, disseminated zoster in immunocompromised patients, secondary bacterial infection, and—uncommonly—encephalitis or stroke (1). The risk of severe and disseminated zoster is markedly elevated among immunocompromised adults, including recipients of haematopoietic stem-cell transplantation, in whom HZ incidence may exceed 30 cases per 1,000 person-years in the absence of vaccination (7).

Two herpes zoster vaccines have been licensed for adult use in the United States. Zostavax (zoster vaccine live, ZVL; Merck & Co.), a single-dose live-attenuated formulation, was licensed in 2006 and demonstrated approximately 51% efficacy against incident HZ, with diminishing effectiveness over time and in older recipients (8, 9). Shingrix (recombinant zoster vaccine, RZV; GlaxoSmithKline), a non-live, adjuvanted glycoprotein E subunit vaccine administered as a two-dose series 2–6 months apart, was licensed in October 2017 and demonstrated approximately 90% efficacy against HZ across age strata, including adults aged 70 years and older (10, 11). Its AS01B adjuvant system drives both the strong VZV-specific cell-mediated and humoral responses that underpin RZV's efficacy and the brisk reactogenicity that has characterized its post-marketing safety profile (12). Real-world evidence from Medicare claims and large integrated health-system cohorts has subsequently confirmed sustained effectiveness of RZV against incident HZ in the years following licensure (13, 14); a post-licensure systematic review and meta-analysis additionally summarized vaccine effectiveness across multiple real-world settings (15); and a placebo-controlled trial in autologous stem-cell transplant recipients demonstrated 68% efficacy in this severely immunocompromised population (7). The Advisory Committee on Immunization Practices (ACIP) accordingly recommends RZV for immunocompetent adults aged 50 years and older (16) and, since 2021, for immunocompromised adults aged 19 years and older (17). Merck ceased US distribution of Zostavax on 18 November 2020, leaving Shingrix as the only herpes zoster vaccine available in the United States. Despite its efficacy, the two-dose schedule of Shingrix and its known reactogenicity have been associated with imperfect completion of the second dose: in a US pharmacy and medical-claims analysis covering more than 200,000 adults, only about three-quarters of recipients received the recommended second dose within the indicated 2–6 month window (18).

Pre-licensure clinical trials are powered to characterize the most common adverse reactions but are typically too small and too short to detect rare or delayed events and to capture the safety experience of populations under-represented in trial enrollment. Post-marketing surveillance is therefore essential. The Vaccine Adverse Event Reporting System (VAERS) is the national passive surveillance system jointly administered by the CDC and the US Food and Drug Administration; established in 1990, VAERS serves as the national early-warning, signal-detection system for adverse events not detected during pre-market testing (19, 20). Reports of adverse events that follow vaccination may be submitted by health-care professionals, manufacturers, vaccine recipients, or family members, regardless of suspected causality; under the National Childhood Vaccine Injury Act of 1986, healthcare providers and manufacturers are required to report a defined set of adverse events to VAERS (19). The strengths of VAERS include nationwide coverage and rapid signal detection; its well-documented limitations include under-reporting of mild events, variable report quality, the absence of denominator data on doses administered, and the lack of an unvaccinated comparison group (20). Sex, age, and other host factors known to modulate the immune response and the propensity to report adverse events further shape the data captured by passive surveillance (21). Long-running evaluations of the VAERS database illustrate both the value of, and the analytic care required by, this type of spontaneous-reporting surveillance (22). Crucially, VAERS is intended for hypothesis generation; it does not on its own establish causality between a vaccine and an observed event (19).

The early post-licensure safety experience of RZV was characterized by Hesse et al. (23) using VAERS data received through June 2018, and a more recent descriptive summary addressed the recombinant vaccine specifically (24). Building on this work, our study spans both licensed herpes zoster vaccines across the full transition from ZVL to RZV using the most recent publicly released VAERS data. Our primary objective was to characterize the post-marketing adverse-event profile of US herpes zoster vaccine reports and to identify events reported disproportionately relative to all other VAERS reports. Secondary objectives were (i) to describe brand-specific reporting patterns for Shingrix and Zostavax across demographics, MedDRA system organ classes and preferred terms, severity indicators, recovery status, and time to onset; and (ii) to examine reporting trends stratified by age, sex, and place of administration.

## 2 Materials and methods

### 2.1 Data source and study design

The unit of analysis throughout this manuscript is the individual VAERS report. We conducted a descriptive cross-sectional analysis of US reports of adverse events after herpes zoster vaccination, reported in accordance with the STROBE (Strengthening the Reporting of Observational Studies in Epidemiology) guidelines for cross-sectional studies; a completed STROBE checklist is provided with the submission, and the RECORD extension for routinely-collected health data was additionally consulted. The publicly released VAERS files for the 8 years 2017 through 2024 were downloaded from vaers.hhs.gov on July 10, 2024. VAERS distributes its data as three linked annual files: a main report file, a vaccine file (one row per administered vaccine), and a symptoms file (with up to five MedDRA preferred terms per report). Each annual file is grouped by the date the report was received; the 2024 file available at the time of analysis contained reports received between 1 January and 25 April 2024. We linked the three files using the de-identified report identifier. Two versions of the VAERS reporting form are accepted by the system: the legacy VAERS 1 form, used by submitters who continued to file paper reports, and the VAERS 2 form, introduced in July 2017 and used for nearly all subsequent submissions. The two forms collect overlapping but not identical information; we describe in the Variables section how fields whose definitions or codes differ between the two forms were harmonized for analysis (19).

### 2.2 Inclusion criteria

Reports were eligible if at least one administered vaccine was coded as a herpes zoster vaccine (VAERS vaccine type “VARZOS,” which is the only zoster category in VAERS). Each report was assigned to a brand on the basis of the vaccine-name field: Shingrix for reports listing “ZOSTER (SHINGRIX),” Zostavax for reports listing “ZOSTER LIVE (ZOSTAVAX),” and “unspecified” for reports listing only “ZOSTER (NO BRAND NAME)”. Reports listing both brands (*n* = 10) were assigned to Shingrix. Consistent with the manuscript's focus on the United States, we restricted the analysis to reports with a recorded US state, the District of Columbia, or a US territory. Reports in which other vaccines had been administered concurrently with the herpes zoster vaccine were retained, because such co-administration reflects routine clinical practice. Of 85,422 reports listing a herpes zoster vaccine during 2017–2024, 15,600 without a recorded US state or territory were excluded, yielding the final cohort of 69,822 US reports; no reports were excluded for missing demographic or clinical fields (Supplementary Figure S1).

### 2.3 Variables

We extracted recipient age in years, recipient sex, vaccine brand, and the place of administration. Sex was coded as recorded by the submitter (Male, Female, or unknown—the latter encompassing both reports coded “U” and reports left blank). The place of administration was grouped from the VAERS source codes into pharmacy, private physician's office, public-health clinic, other facility (e.g., long-term care, occupational, hospital outpatient), and a residual “other/unknown” category that pooled the smaller settings (military, workplace, school, senior-living facility, and reports for which the place of administration was not specified). Several of these place-of-administration codes—pharmacy, school, senior-living facility, and workplace—are available only on the VAERS 2 form; the small minority of reports submitted on the legacy VAERS 1 form (*n* = 3,516, 5.0% of the cohort) cannot be classified into those specific settings and are captured in the residual “other/unknown” category for this purpose (19).

Each report's adverse events were taken from up to five MedDRA preferred-term fields and aggregated to the report level, so that a given preferred term was counted at most once per report. Severity was classified using the five binary indicators that VAERS captures for the US Food and Drug Administration's serious-adverse-event criteria (death, life-threatening event, hospitalization, prolongation of an existing hospitalization, and permanent disability); a report was considered serious if any of these indicators was present. Recovery status was recorded as recovered, not recovered, unknown, or missing. The time from vaccination to onset of the first reported symptom was taken as the number of days reported by the submitter and, for descriptive summaries, restricted to plausible values of 0–365 days.

### 2.4 Statistical analysis

Categorical variables are presented as counts and percentages, with the denominator indicated for each table (overall cohort, brand subgroup, age stratum, sex stratum, or place-of-administration stratum). Variables with incomplete reporting—including age and sex—were retained and summarized for every report with a recorded value; the proportion missing is shown explicitly for each variable rather than excluded, and missing values were not imputed; because the missingness of key fields (notably age) is strongly differential by brand and reporting era and is therefore unlikely to be missing at random, we present complete-case summaries with the missing fraction shown explicitly and report a sensitivity analysis of the age strata (Supplementary Table S5). Continuous variables are summarized as the median and interquartile range; for age we additionally report the mean and standard deviation. Because spontaneous reports lack a denominator and an unvaccinated comparator and are subject to differential reporting, we did not perform formal significance tests comparing Shingrix with Zostavax; the two brand subgroups are described using counts and percentages only.

To identify adverse events reported disproportionately often for herpes zoster vaccines as a group, we computed the reporting odds ratio (ROR), a standard pharmacovigilance measure of disproportionate reporting (25). For each event we built a 2 × 2 table cross-classifying report source—the combined herpes zoster vaccine cohort versus all other US VAERS reports received during 2017–2024 (the conventional background reference for disproportionality analysis, representing expected reporting across the surveillance system)—by the presence or absence of the event, calculated the ROR as the odds of the event in the herpes zoster vaccine cohort divided by the corresponding odds in the comparator reports, and computed 95% confidence intervals on the logarithmic scale. When any cell of the 2 × 2 table contained a zero count, 0.5 was added to all four cells, a standard continuity adjustment. An ROR greater than 1 indicates that the event was reported proportionately more often for herpes zoster vaccines than for other vaccines in VAERS; the measure is a signal-detection tool and is not a comparison between Shingrix and Zostavax. We screened the herpes zoster vaccine category as a whole against the full VAERS background because both vaccines share the same structured VAERS vaccine-type code (VARZOS) and clinical indication, and a class-level comparator maximizes stability for low-frequency preferred terms; between-brand differences are conveyed descriptively in Tables 1, 2A, 2B and 3. As an exploratory addition, we computed brand-specific RORs (each brand versus the same all-other-VAERS comparator, not against each other) restricted to reactogenicity and systemic preferred terms (Supplementary Table S3); the 502 reports with an unspecified brand are included in the combined-cohort exposed group but are excluded from the brand-stratified exposed groups. ROR estimates are sensitive to the composition of the comparator, which over 2017–2024 was dominated by COVID-19 vaccine reports (see Results and Limitations). As with any disproportionality measure derived from passive surveillance, RORs do not estimate relative risk and cannot be interpreted causally. Consistent with standard pharmacovigilance practice, RORs are reported unadjusted; because VAERS lacks a denominator of doses administered and an unvaccinated comparator, disproportionality measures are not risk estimates and formal adjustment for confounders such as age or sex is not applicable, although recipient age, sex, brand, and calendar period are correlated and should temper interpretation of the descriptive strata. All preferred-term and system organ class percentages are report-based: the numerator is the number of reports listing the term (each term counted once per report) and the denominator is the total number of reports in the relevant column, not the number of events. All analyses were conducted using Stata version 16.1 (StataCorp LLC, College Station, TX, USA).

**Table 1:** Demographic characteristics of US herpes zoster vaccine VAERS reports, 2017–2024.

Characteristic	Overall (*N* = 69,822)	Shingrix (n = 58,813)	Zostavax (n = 10,507)
Age (years), *n* with known age	51,636	48,397	3,043
Mean ± SD	64.4 ± 9.8	64.6 ± 9.6	62.3 ± 12.1
Median (IQR)	64.0 (58.0–71.0)	64.0 (58.0–71.0)	63.0 (60.0–68.0)
Age group, *n* (% of column)			
18–49	670 (1.0%)	525 (0.9%)	139 (1.3%)
50–59	15,084 (21.6%)	14,460 (24.6%)	543 (5.2%)
60–69	20,778 (29.8%)	18,963 (32.2%)	1,763 (16.8%)
70–79	11,865 (17.0%)	11,338 (19.3%)	489 (4.7%)
≥80	3,239 (4.6%)	3,111 (5.3%)	109 (1.0%)
Unknown	18,186 (26.0%)	10,416 (17.7%)	7,464 (71.0%)
Sex, *n* (% of column)			
Female	44,605 (63.9%)	38,814 (66.0%)	5,585 (53.2%)
Male	19,169 (27.5%)	16,539 (28.1%)	2,529 (24.1%)
Unknown/undifferentiated	6,048 (8.7%)	3,460 (5.9%)	2,393 (22.8%)
Place of administration, *n* (% of column)			
Pharmacy	33,194 (47.5%)	29,839 (50.7%)	3,288 (31.3%)
Private/physician's office	8,784 (12.6%)	7,418 (12.6%)	1,318 (12.5%)
Other facility	4,923 (7.1%)	2,201 (3.7%)	2,697 (25.7%)
Public health clinic	1,335 (1.9%)	1,109 (1.9%)	220 (2.1%)
Other/Unknown‡	21,586 (30.9%)	18,246 (31.0%)	2,984 (28.4%)

SD, standard deviation; IQR, interquartile range. Percentages are calculated against the column total (overall N or brand n). The Shingrix and Zostavax columns are descriptive; no formal between-brand significance tests were performed. ‡ “Other/Unknown” pools the smaller administration-place categories (military, workplace, school, senior-living facility) together with reports for which the place of administration was not recorded. The Shingrix and Zostavax columns do not sum to Overall because 502 reports (0.7%) with an unspecified brand are included in Overall but not shown as a separate brand column.

**Table 2A:** Frequency of reported MedDRA preferred terms grouped by system organ class (systemic and injection-site classes), US herpes zoster vaccine VAERS reports, 2017–2024.

MedDRA system organ class/preferred term	Overall *n* (%)	Shingrix *n* (%)	Zostavax *n* (%)
**General disorders and administration-site conditions**	**40,241 (57.6%)**	**37,272 (63.4%)**	**2,741 (26.1%)**
Pyrexia	12,746 (18.3%)	12,474 (21.2%)	194 (1.8%)
Pain	12,408 (17.8%)	10,951 (18.6%)	1,369 (13.0%)
Chills	11,114 (15.9%)	10,959 (18.6%)	97 (0.9%)
Injection–site pain	9,925 (14.2%)	9,559 (16.3%)	333 (3.2%)
Fatigue	9,151 (13.1%)	8,985 (15.3%)	114 (1.1%)
Injection–site erythema	7,801 (11.2%)	7,126 (12.1%)	655 (6.2%)
Injection–site swelling	5,518 (7.9%)	5,055 (8.6%)	448 (4.3%)
Erythema	4,763 (6.8%)	4,404 (7.5%)	335 (3.2%)
Influenza–like illness	3,479 (5.0%)	3,432 (5.8%)	34 (0.3%)
Malaise	2,943 (4.2%)	2,863 (4.9%)	59 (0.6%)
Asthenia	2,798 (4.0%)	2,699 (4.6%)	79 (0.8%)
**Musculoskeletal and connective–tissue disorders**	**16,542 (23.7%)**	**16,113 (27.4%)**	**345 (3.3%)**
Pain in extremity	8,897 (12.7%)	8,669 (14.7%)	185 (1.8%)
Myalgia	5,960 (8.5%)	5,857 (10.0%)	69 (0.7%)
Arthralgia	3,509 (5.0%)	3,402 (5.8%)	88 (0.8%)
Back pain	1,146 (1.6%)	1,085 (1.8%)	56 (0.5%)
**Nervous system disorders**	**15,231 (21.8%)**	**14,693 (25.0%)**	**460 (4.4%)**
Headache	10,766 (15.4%)	10,472 (17.8%)	240 (2.3%)
Dizziness	3,093 (4.4%)	2,968 (5.0%)	108 (1.0%)
Paraesthesia	1,273 (1.8%)	1,176 (2.0%)	91 (0.9%)
**Gastrointestinal disorders**	**7,795 (11.2%)**	**7,600 (12.9%)**	**153 (1.5%)**
Nausea	6,100 (8.7%)	5,968 (10.1%)	106 (1.0%)
Vomiting	1,558 (2.2%)	1,505 (2.6%)	40 (0.4%)
Diarrhea	1,549 (2.2%)	1,506 (2.6%)	27 (0.3%)

SOC, system organ class; PT, preferred term. Values are the number (%) of reports listing the term; each preferred term is counted at most once per report, and percentages are calculated against the column report total (Overall 69,822; Shingrix 58,813; Zostavax 10,507), not the number of events. Because a report may list several preferred terms within one system organ class, an SOC count is the number of reports with at least one preferred term in that class and need not equal the sum of the individual preferred–term counts (only the most frequently reported preferred terms within each class are displayed); no report is counted more than once at the SOC level. The Shingrix and Zostavax columns do not sum to Overall because 502 reports (0.7%) with an unspecified brand are included in Overall but not shown as a separate brand column.

**Table 2B:** Frequency of reported MedDRA preferred terms grouped by system organ class (dermatological, infective, and immune classes), US herpes zoster vaccine VAERS reports, 2017–2024.

MedDRA system organ class/preferred term	Overall *n* (%)	Shingrix *n* (%)	Zostavax *n* (%)
**Skin and subcutaneous–tissue disorders**	**13,078 (18.7%)**	**9,251 (15.7%)**	**3,755 (35.7%)**
Rash	5,860 (8.4%)	4,941 (8.4%)	883 (8.4%)
Rash vesicular	3,275 (4.7%)	609 (1.0%)	2,658 (25.3%)
Pruritus	3,129 (4.5%)	2,837 (4.8%)	260 (2.5%)
Urticaria	1,735 (2.5%)	1,634 (2.8%)	88 (0.8%)
**Infections and infestations**	**9,799 (14.0%)**	**3,234 (5.5%)**	**6,478 (61.7%)**
Herpes zoster	9,772 (14.0%)	3,215 (5.5%)	6,470 (61.6%)
Vaccination failure	964 (1.4%)	648 (1.1%)	260 (2.5%)
**Immune–system disorders**	**482 (0.7%)**	**428 (0.7%)**	**43 (0.4%)**
Hypersensitivity	422 (0.6%)	376 (0.6%)	36 (0.3%)
Anaphylactic reaction	63 (0.09%)	54 (0.09%)	8 (0.08%)

SOC, system organ class; PT, preferred term. Values are the number (%) of reports listing the term; each preferred term is counted at most once per report, and percentages are calculated against the column report total (Overall 69,822; Shingrix 58,813; Zostavax 10,507), not the number of events. Because a report may list several preferred terms within one system organ class, an SOC count is the number of reports with at least one preferred term in that class and need not equal the sum of the individual preferred–term counts (only the most frequently reported preferred terms within each class are displayed); no report is counted more than once at the SOC level. The Shingrix and Zostavax columns do not sum to Overall because 502 reports (0.7%) with an unspecified brand are included in Overall but not shown as a separate brand column.

**Table 3:** Severity indicators, recovery status, and time to symptom onset, US herpes zoster vaccine VAERS reports, 2017–2024.

Indicator/outcome	Overall (*N* = 69,822)	Shingrix (*n* = 58,813)	Zostavax (*n* = 10,507)
Serious report (any FDA criterion)	4,597 (6.6%)	1,681 (2.9%)	2,899 (27.6%)
Hospitalization	2,811 (4.0%)	1,116 (1.9%)	1,682 (16.0%)
Permanent disability	1,950 (2.8%)	606 (1.0%)	1,339 (12.7%)
Life–threatening event	334 (0.48%)	228 (0.39%)	106 (1.01%)
Death	144 (0.21%)	73 (0.12%)	69 (0.66%)
Prolongation of hospitalization	12 (0.02%)	6 (0.01%)	6 (0.06%)
Recovery status			
Recovered/recovering	20,763 (29.7%)	19,896 (33.8%)	764 (7.3%)
Not recovered at reporting	23,416 (33.5%)	19,361 (32.9%)	3,929 (37.4%)
Unknown	22,390 (32.1%)	16,490 (28.0%)	5,658 (53.8%)
Missing	3,253 (4.7%)	3,066 (5.2%)	156 (1.5%)
Time to onset (days), *n* with valid value	48,184	45,299	2,700
Median (IQR)	1 (0–1)	1 (0–1)	1 (0–3)
Within 0–3 days, *n* (% of valid)	42,534 (88.3%)	40,251 (88.9%)	2,130 (78.9%)
4–7 days	2,191 (4.5%)	2,046 (4.5%)	140 (5.2%)
8–14 days	1,185 (2.5%)	1,077 (2.4%)	99 (3.7%)
15–30 days	893 (1.9%)	799 (1.8%)	86 (3.2%)
>30 days	1,381 (2.9%)	1,126 (2.5%)	245 (9.1%)

IQR, interquartile range. Percentages are calculated against the column total unless noted. Severity criteria can co–occur; the sum of individual criteria exceeds the count of reports meeting any criterion. The Shingrix and Zostavax columns are descriptive; no formal between-brand significance tests were performed. The VAERS death indicator marks reports with a fatal outcome and does not denote a vaccine-attributed or verified cause of death. The Shingrix and Zostavax columns do not sum to Overall because 502 reports (0.7%) with an unspecified brand are included in Overall but not shown as a separate brand column.

### 2.5 Ethics

This study analyzed the publicly available, fully de-identified US VAERS public-use dataset and did not involve human participants, identifiable human data, or human tissue. Under the US Common Rule (45 CFR 46.102), the secondary analysis of these de-identified data did not constitute human-subjects research and did not require institutional review board approval or a waiver. The study was conducted in accordance with the Declaration of Helsinki (2013 revision); no attempt was made to re-identify reporters or vaccine recipients.

## 3 Results

### 3.1 Annual reporting volume and vaccine type

**Figure 1:**
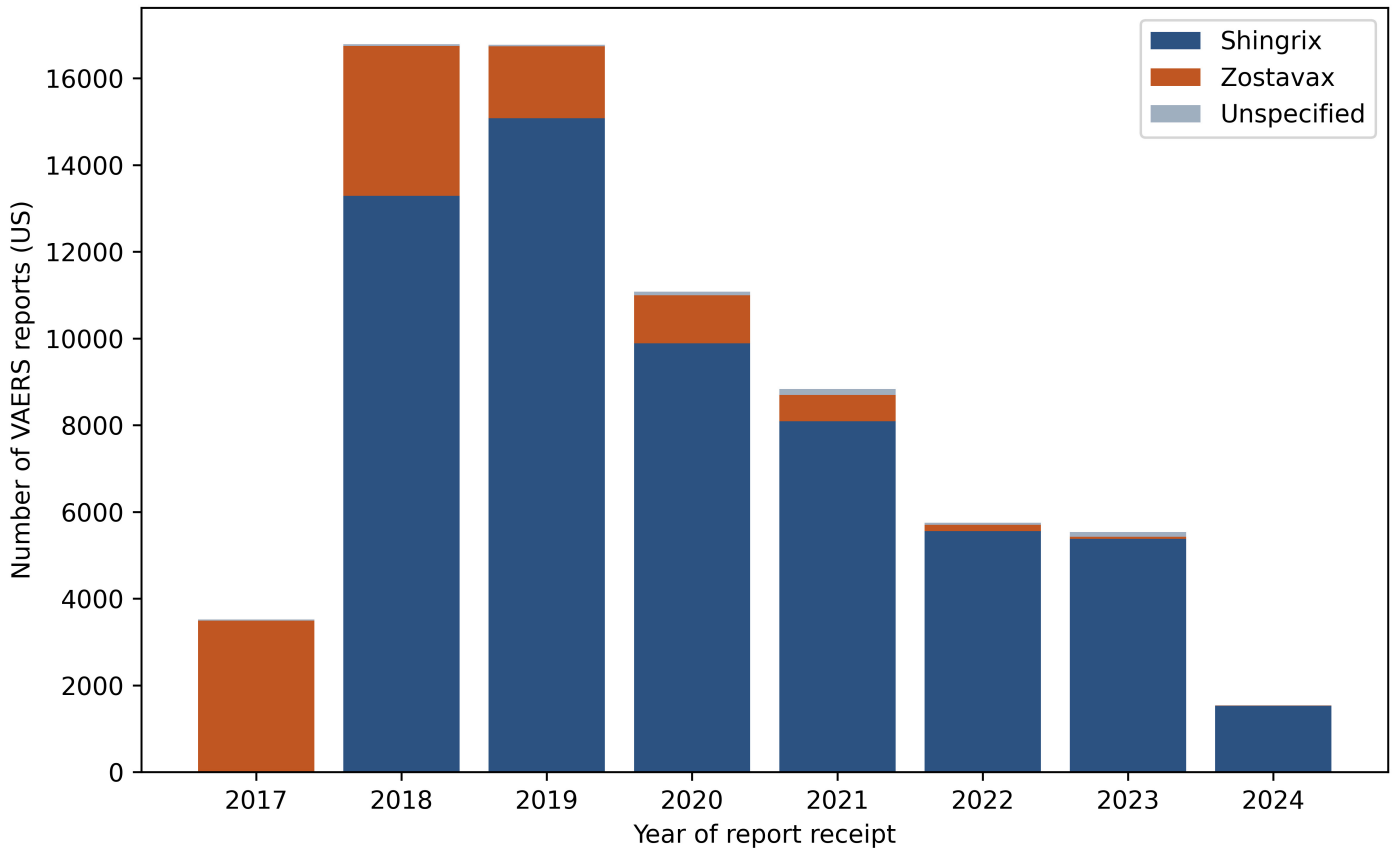
Annual US VAERS reports for herpes zoster vaccines by brand, 2017–2024. The 2024 bar reflects only reports received through 25 April 2024.

Between 1 January 2017 and 25 April 2024, VAERS received 69,822 US reports listing a herpes zoster vaccine. Shingrix was specified in 58,813 reports (84.2%), Zostavax in 10,507 (15.0%), and the brand was not specified in 502 (0.7%). Annual volume rose sharply after Shingrix became available in late 2017, peaking in 2018 (16,785 reports) and 2019 (16,774 reports), and then declined progressively to 5,537 reports in 2023, with 1,547 reports received in the first ~4 months of 2024 (Figure 1, Supplementary Table S1). The early-period increase is consistent with the well-documented Weber effect, in which adverse-event reporting peaks within the first few years following the introduction of a new vaccine or therapeutic product before declining toward a stable baseline (26). The subsequent decline is likely multifactorial: in addition to the Weber effect, it is consistent with a genuine reduction in doses administered as the age-eligible population became increasingly vaccinated (market saturation) and with waning media and clinical attention over the product lifecycle. Because VAERS lacks denominator data, a change in reporting behavior cannot be distinguished from a true decline in vaccine administration. Zostavax reports declined steeply following Merck's withdrawal of US supply in late 2020, falling from 1,656 in 2019 to 5 in 2024. Another vaccine was co-administered with the herpes zoster vaccine in 5,199 reports (7.4%; 7.9% of Shingrix and 4.7% of Zostavax reports), most commonly influenza, pneumococcal, and tetanus-containing vaccines; because reactogenicity from concurrent vaccines cannot be disentangled in passive data, co-administered reports may contribute to the reported reactogenicity profile, though at 7.4% they are unlikely to materially drive it.

### 3.2 Demographic characteristics

**Figure 2:**
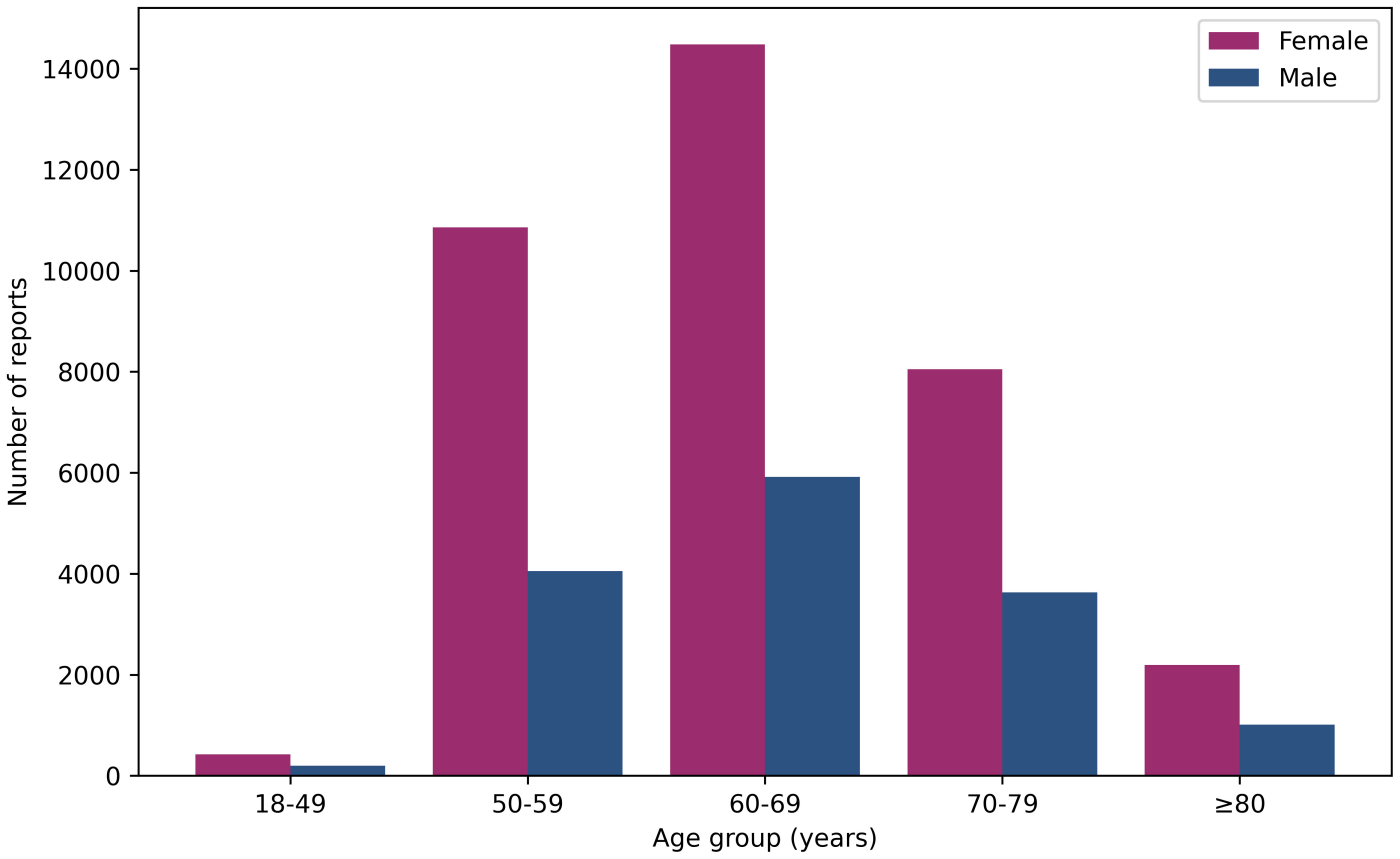
Age and sex distribution of US herpes zoster vaccine VAERS reports with both values documented, 2017–2024.

Demographic characteristics are summarized in Table 1, Figure 2. Among the 51,636 reports with a documented recipient age, the median age was 64.0 years (IQR 58.0–71.0; mean 64.4 years, SD 9.8). Across the cohort, 670 reports (1.0%) involved adults aged 18–49 years, 15,084 (21.6%) aged 50–59, 20,778 (29.8%) aged 60–69, 11,865 (17.0%) aged 70–79, and 3,239 (4.6%) aged ≥80 years; age was missing in 18,186 reports (26.0%). Median age was slightly higher for Shingrix recipients (64.0 years, IQR 58.0–71.0) than for Zostavax recipients (63.0 years, IQR 60.0–68.0). Women accounted for 44,605 reports (63.9% of the cohort and 69.9% of reports with a known sex), men for 19,169 (27.5%), and sex was missing or undifferentiated in 6,048 (8.7%). The female predominance was modestly stronger among Shingrix reports (38,814 of 55,353 with known sex, 70.1%) than among Zostavax reports (5,585 of 8,114, 68.8%).

### 3.3 Reported adverse events

**Figure 3:**
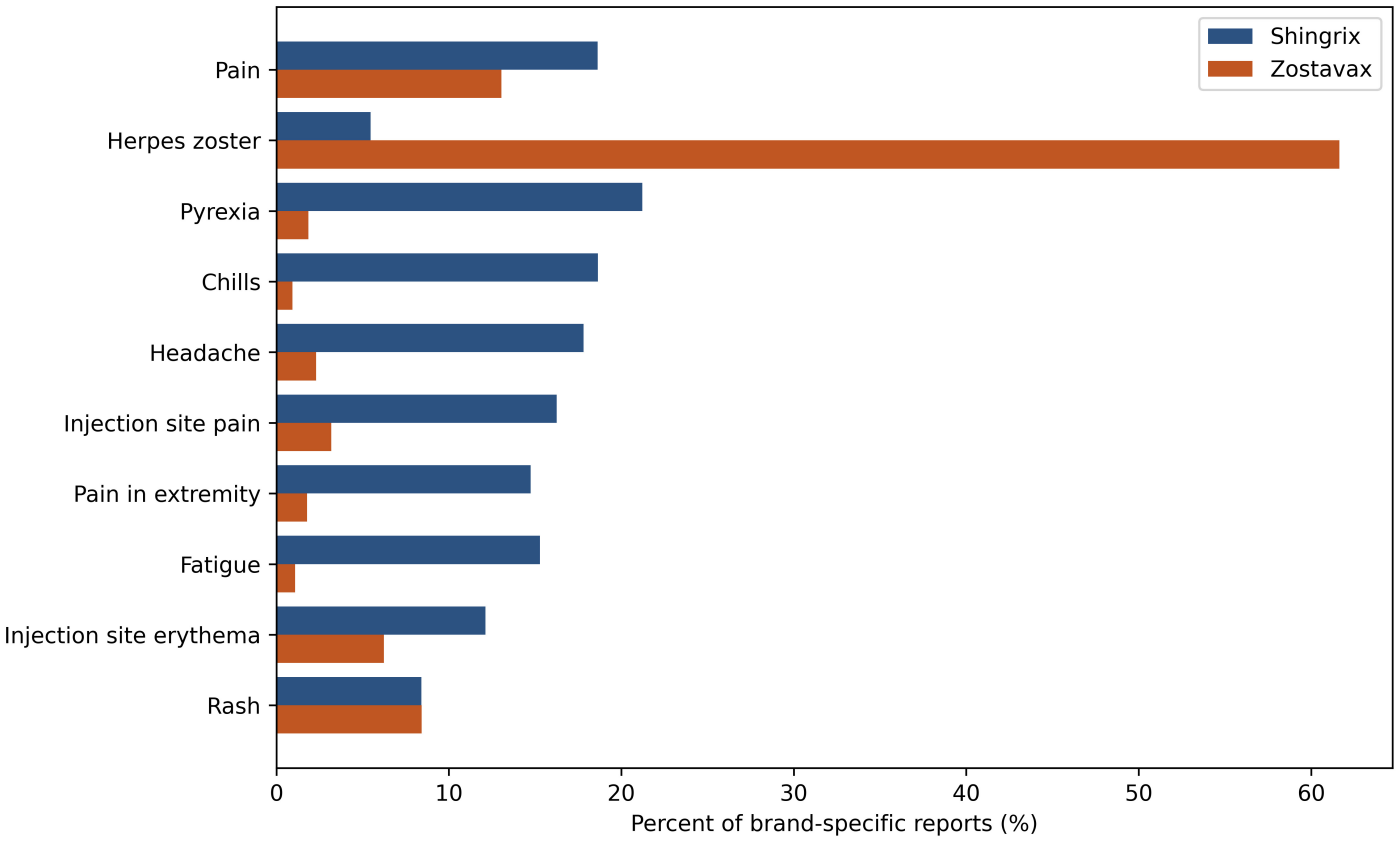
Ten most frequent MedDRA preferred terms in US herpes zoster vaccine VAERS reports, displayed as the percentage of brand-specific reports for Shingrix and Zostavax.

Across the 69,822 US reports, the most frequently reported MedDRA preferred terms (PTs) were pyrexia (12,746 reports; 18.3%), pain (12,408; 17.8%), chills (11,114; 15.9%), headache (10,766; 15.4%), injection-site pain (9,925; 14.2%), herpes zoster (9,772; 14.0%), fatigue (9,151; 13.1%), pain in extremity (8,897; 12.7%), injection-site erythema (7,801; 11.2%), and nausea (6,100; 8.7%); the corresponding brand-specific frequencies are shown in Figure 3. Reactogenic constitutional symptoms and injection-site reactions were prominent, consistent with the adjuvanted formulation of Shingrix and the known reactogenicity profile observed in pre-licensure trials (10, 11). The most frequently reported preferred terms, grouped into MedDRA system organ classes, are presented in Tables 2A and 2B.

Relative to all other US VAERS reports received during 2017–2024 [*n* = 1,029,498, of which 839,855 (81.6%) were COVID-19 vaccine reports], several events were reported disproportionately often for herpes zoster vaccines. Injection-site reactions and influenza-like illness were enriched (injection-site pain ROR 2.92, 95% CI 2.86–2.99; injection-site erythema 2.78, 2.71–2.85; injection-site swelling 2.31, 2.24–2.38; influenza-like illness 4.55, 4.37–4.73), consistent with the reactogenicity expected of an adjuvanted vaccine. The strongest disproportionality signal was vesicular rash (ROR 43.70, 40.85–46.75), which the brand-stratified counts show was concentrated among Zostavax reports (herpes zoster in 61.6% of Zostavax versus 5.5% of Shingrix reports; vesicular rash in 25.3% versus 1.0%), consistent with the live-attenuated formulation. This extreme value almost certainly reflects protopathic bias (confounding by indication): vesicular rash after the live-attenuated vaccine represents reported vaccine-strain or breakthrough disease—an expected manifestation of a live vaccine's biology—rather than an unexpected adverse-event signal or a measure of risk. For the same reason, and because a report of herpes zoster after a herpes zoster vaccine cannot be distinguished from wild-type or breakthrough disease, we did not compute a herpes zoster ROR. Brand-specific RORs restricted to reactogenicity and systemic terms are given in Supplementary Table S3. For Shingrix, point estimates were slightly higher than for the combined cohort (e.g., injection-site pain ROR 3.42; influenza-like illness ROR 5.37), consistent with Shingrix accounting for most reactogenicity reports. For Zostavax, by contrast, most reactogenicity terms were reported proportionately less often than in the comparator (e.g., chills ROR 0.09; influenza-like illness ROR 0.28), consistent with a live vaccine whose reports are dominated by zoster-family events rather than by reactogenicity. By contrast, several common systemic symptoms were not disproportionately reported for herpes zoster vaccines relative to other vaccines (e.g., fatigue ROR 1.13, headache 1.18, nausea 1.15; gastrointestinal disorders as a class 0.99), and anaphylactic reaction was reported less often ROR 0.41.

### 3.4 Severity indicators and outcomes

Severity indicators and outcomes are summarized in Table 3. Across the cohort, 4,597 reports (6.6%) met FDA criteria for a serious adverse event, with hospitalization being the most common qualifying criterion (2,811 reports; 4.0% of the cohort), followed by permanent disability (1,950; 2.8%), life-threatening event (334; 0.48%), death (144; 0.21%), and prolongation of an existing hospitalization (12; 0.02%). Multiple criteria could be flagged on a single report. The preferred term Guillain-Barré syndrome was recorded in 194 reports (0.28% of the cohort; 168 Shingrix, 23 Zostavax, 3 unspecified brand); because VAERS captures at most five preferred terms per report and provides no denominator, this is a raw report count and a lower bound rather than an incidence estimate.

Descriptively, the proportion of reports meeting serious-event criteria was higher for Zostavax (2,899 of 10,507; 27.6%) than for Shingrix (1,681 of 58,813; 2.9%). This 27.6% is a proportion of reports, not an incidence or a risk, as VAERS provides no denominator of doses administered. It corresponds to a larger share of Zostavax reports describing hospitalization and disabling sequelae of reported herpes zoster following the live-attenuated vaccine (hospitalization 16.0% vs. 1.9%; permanent disability 12.7% vs. 1.0%); because serious manifestations reported after a live vaccine are inherently more likely to be reported and to meet a serious criterion, this proportion reflects the composition of what is reported for a live vaccine rather than a true excess risk of serious adverse events. A death was recorded in 144 reports overall (0.21%); the proportion of reports recording a death was higher for Zostavax (69 reports; 0.66%) than for Shingrix (73 reports; 0.12%), although absolute numbers were small. The VAERS death indicator marks reports with a fatal outcome and does not denote a vaccine-attributed or verified cause of death; deaths reported after Zostavax more often involved older recipients, longer surveillance follow-up, and a higher baseline risk of severe outcomes among older or immunocompromised recipients of a live vaccine.

Time from vaccination to symptom onset was reported for 48,184 reports (69.0% of the cohort) within the plausible range of 0–365 days. The median onset interval was 1 day (IQR 0–1), and 88.3% of reports with a known interval recorded an onset within 3 days of vaccination. Onset was slightly later for Zostavax (median 1 day, IQR 0–3) than for Shingrix (median 1 day, IQR 0–1), reflecting both reactogenicity timing for Shingrix and the longer interval to reported herpes zoster following Zostavax. Recovery status was documented as recovered/recovering in 20,763 reports (29.7% of cohort; 47.0% of those with a known status) and as not recovered at the time of reporting in 23,416 (33.5%; 53.0% of those with a known status). Recovery status was marked Unknown in 22,390 reports (32.1%) and was missing in a further 3,253 (4.7%); these high proportions of indeterminate recovery status reflect the limited follow-up captured in passive surveillance and should temper any inference about durable outcomes.

### 3.5 Stratified analysis

Stratified report counts and the proportion meeting serious-event criteria are shown in Supplementary Table S2. Among reports with documented age, the largest single age stratum was 60–69 years (20,778; 29.8% of cohort, 40.2% of reports with known age), followed by 50–59 (15,084; 21.6%) and 70–79 (11,865; 17.0%). The proportion of reports classified as serious increased monotonically with age, from 1.9% in adults 18–49 years and 2.7% in those 50–59 years to 5.1% in those ≥80 years. Although VAERS is a passive system without the denominator data needed to compare age-specific risks formally, the higher proportional burden of serious reports in older recipients is consistent with their greater baseline vulnerability to severe disease and complications.

Reports involving women outnumbered those involving men by approximately 2.3 to 1 (44,605 vs. 19,169 with documented sex), but a higher proportion of men's reports met serious criteria than women's (7.6% vs. 5.1%). Reports were most often linked to administration in a pharmacy (33,194; 47.5% of cohort), followed by a private physician's office (8,784; 12.6%) and other facilities (4,923; 7.1%); place of administration was either unspecified or recorded as one of the smaller residual categories (military, workplace, school, or senior-living facility) for 21,586 reports (30.9%). The proportion of serious reports varied by setting, being lowest in pharmacy reports (5.3%) and highest, among the grouped strata shown in Table 1, in the “other facility” category (9.8%); among the smaller settings itemized in Supplementary Table S4, military (13.4%) and senior-living (13.0%) reports had higher proportions, on very small counts, with intermediate proportions in the residual “other/unknown” stratum (7.8%) and in private physician's offices (7.0%); these between-setting differences should be interpreted cautiously because place of administration is correlated with vaccine brand, calendar period, and recipient case-mix. Because place of administration is a single coded field, the heterogeneous “other facility” category (e.g., long-term care, occupational, hospital outpatient) cannot be decomposed further from the structured data; a full breakdown of all recorded settings with serious-report proportions is provided in Supplementary Table S4. The higher serious-report proportion in this stratum likely reflects case-mix, including the marked over-representation of the live Zostavax in that setting (25.7% vs. 3.7% of Shingrix reports).

## 4 Discussion

### 4.1 Principal findings

This analysis of 69,822 US VAERS reports submitted between January 2017 and April 2024 yields four principal findings. First, the post-licensure adverse-event profile of the recombinant zoster vaccine Shingrix is dominated by reactogenic constitutional symptoms (pyrexia, chills, fatigue, headache, myalgia) and injection-site reactions, consistent with the adjuvanted formulation and with the reactogenicity observed in the ZOE-50 and ZOE-70 pivotal trials (10, 11) and in the early post-licensure VAERS analysis by Hesse et al. (23). Second, the live-attenuated vaccine Zostavax shows a distinctly different reporting profile dominated by reported herpes zoster, vesicular rash, and disseminated cutaneous events (not virologically confirmed as vaccine-strain); herpes zoster was reported in 61.6% of Zostavax reports compared with 5.5% of Shingrix reports, and vesicular rash in 25.3% versus 1.0%, and vesicular rash was strongly over-represented in the combined herpes zoster vaccine cohort relative to all other US VAERS reports (ROR 43.70, 95% CI 40.85–46.75). Third, serious-event reports as defined by FDA criteria were uncommon for Shingrix (2.9% of Shingrix reports) but substantially more common in absolute terms for Zostavax (27.6%), reflecting a larger number of reports describing hospitalized and disabling cases of herpes zoster reported after the live-attenuated vaccine, particularly in older adults. Fourth, annual report volume rose sharply during 2018–2019 in parallel with Shingrix uptake and declined progressively thereafter, a pattern consistent with the Weber effect commonly observed in passive surveillance after the introduction of a new vaccine (26).

Across the entire VAERS database, approximately 85%–90% of reports describe relatively minor events such as fevers or local injection-site reactions, with fewer than 15% describing serious outcomes such as hospitalization, life-threatening illness, or death (19). The 6.6% serious-report proportion observed in the present herpes zoster vaccine cohort is therefore lower than the all-vaccine VAERS benchmark. The brand-stratified figures show that this favorable overall proportion reflects the Shingrix subset (2.9% serious), while the Zostavax subset (27.6%) sits well above the all-vaccine benchmark—a disparity that is consistent with the live-attenuated nature of Zostavax and with the over-representation of severe herpes zoster reported after the live vaccine among its VAERS reports rather than with a generalized safety concern about the recombinant vaccine.

### 4.2 Comparison with prior surveillance

Our analysis extends the early post-licensure surveillance summary by Hesse et al. (23), which characterized US VAERS reports received between October 2017 and June 2018—the first ~9 months following Shingrix licensure. Hesse et al. reported 4,381 RZV adverse-event reports during that interval, of which 130 (3.0%) met criteria for a serious event. The qualitative pattern they reported—predominantly non-serious reactogenic events with infrequent serious outcomes—is reproduced here using the extended 2017–2024 dataset: 13,293 US Shingrix reports were identified during calendar years 2017–2018 in the present analysis; across the full eight-year cohort, 2.86% of Shingrix reports met FDA serious-event criteria, closely matching the 3.0% serious-event proportion Hesse et al. reported for their shorter interval. The extended observation period further demonstrates that the early Shingrix reporting peak has not been sustained: annual Shingrix reports declined from 15,079 in 2019 to 5,378 in 2023, paralleling both the maturation of the product lifecycle and the decline in age-eligible primary uptake. The Zostavax reporting trajectory mirrors Merck's market withdrawal in late 2020, with only 5 Zostavax reports received in the first ~4 months of 2024. Two further post-marketing safety analyses bracket the surveillance interval examined here. The manufacturer's pharmacovigilance review by Tavares-Da-Silva et al. (27) summarized 15,636 spontaneous adverse-event reports submitted directly to GSK from approximately 9.3 million Shingrix doses distributed worldwide through February 2019, of which 95.3% were classified as non-serious; the demographic distribution (50–69 years, 62.1%; female, 66.7%) closely mirrors that observed in the present US-resident cohort. More recently, Shu et al. (24) analyzed 66,849 VAERS reports for Shingrix received between October 2017 and April 2024—a period that overlaps almost exactly with the analytic window used here — of which 97.3% were classified as non-serious (their figure of 1,279,596 refers to all vaccine reports received by VAERS over that interval, from which the Shingrix reports were drawn). Our analysis is complementary but distinct: whereas Shu et al. characterized the recombinant vaccine alone, we describe both licensed herpes zoster vaccines across the full Zostavax-to-Shingrix transition, add a disproportionality (ROR) analysis against all other VAERS reports, and provide brand-stratified severity, onset, recovery, and place-of-administration profiles. The Shingrix report counts are of similar magnitude (66,849 vs. 58,813 here), the difference reflecting our restriction to US-resident reports and the VARZOS structured case definition, and the convergence across these independent analyses reinforces the robustness of the predominantly non-serious adverse-event profile.

Active-surveillance studies in healthcare-claims and electronic-health-record systems have characterized the rare-but-important serious-outcome profile of Shingrix that passive surveillance cannot quantify. Using Medicare claims data, Goud et al. (28) identified a small but statistically detectable excess risk of Guillain-Barré syndrome (GBS) of approximately three cases per million doses within 42 days of vaccination, while concluding that the overall benefit–risk balance remained in favor of vaccination. In response to this signal, the US Food and Drug Administration added a boxed warning regarding the possible GBS risk to the Shingrix prescribing information in March 2021 (29). In the present cohort, Guillain-Barré syndrome was recorded in 194 reports (Results); consistent with the passive-surveillance caveats noted above, this raw count cannot be converted to an incidence and is not comparable to the denominator-based Medicare estimate of Goud et al.; subsequent active-surveillance work has not detected an escalating signal. A complementary Vaccine Safety Datalink active-surveillance study by Nelson et al. (30) monitored ~648,000 RZV doses administered in 2018–2019 and identified no sustained increased risks for the prespecified outcomes examined, and a separate broad safety assessment by Yih et al. (31) applying data-mining methods to 1,014,329 RZV doses likewise detected no signals beyond the established reactogenicity profile. Because VAERS lacks denominator data and an unvaccinated comparator, the present descriptive analysis cannot quantify absolute risk and is intended only to characterize post-marketing reporting patterns. The disproportionate reporting of specific events for herpes zoster vaccines, and the descriptive differences between the two brands, should be interpreted as reflecting differences in vaccine biology (recombinant adjuvanted vs. live-attenuated), differences in indicated populations and clinical use over time, and differences in reporting behavior following the introduction of a new product.

### 4.3 Strengths

Strengths of the present analysis include the eight-year observation window spanning from Shingrix licensure through the post-Zostavax-withdrawal period, the uniform case definition based on the VAERS structured vaccine-type code (which avoids the ambiguity introduced by free-text searches of vaccine names), the explicit use of MedDRA preferred terms grouped into system organ classes, and the transparent treatment of missing data. All summary statistics are reproduced from the publicly available VAERS files using a documented analytic pipeline.

### 4.4 Limitations

Limitations are those inherent to passive surveillance. First, VAERS is a hypothesis-generating system; reports do not establish causation, and signals identified by disproportionality analysis must be confirmed using active-surveillance designs (19). Second, completeness varies across fields: 26.0% of reports lacked recipient age, 8.7% lacked sex, 29.8% lacked a specified place of administration (the 30.9% shown in Table 1 additionally pools the small military, workplace, school, and senior-living categories; Supplementary Table S4), and 32.1% had recovery status coded as unknown with an additional 4.7% missing. We display all missing categories explicitly rather than imputing, but this missingness limits stratified inference. Age missingness was strongly differential (71.0% for Zostavax versus 17.7% for Shingrix) and was associated with the earlier reporting era and the legacy VAERS-1 form, indicating a non-random pattern; we therefore did not impute age. A sensitivity analysis comparing reports with and without a documented age (Supplementary Table S5) shows that this missingness is informative and differential by brand: the serious-report proportion was 3.5% among reports with a documented age versus 15.4% among those without, a contrast that reflects the concentration of Zostavax reports in the missing-age group. The age-stratified results are therefore complete-case analyses, are most representative of the Shingrix, VAERS-2-era cohort, and no analysis can establish how the 26.0% of reports lacking age would alter the observed gradient. Third, the disproportionality (ROR) analysis compares reporting for herpes zoster vaccines with reporting for all other vaccines in VAERS and is therefore sensitive to the composition of the comparator database—which over the study period was dominated by COVID-19 vaccine reports (839,855 of 1,029,498; 81.6%)—as well as to differential reporting behavior and to the well-described notoriety effect that follows the launch of a new vaccine; RORs are crude, unadjusted signals of disproportionate reporting rather than measures of risk, and are not adjusted for age, sex, calendar period, or comparator composition; signals for zoster-family terms (herpes zoster, vesicular rash) reflect vaccine-strain or breakthrough disease and protopathic bias rather than adverse reactions. Fourth, this analysis is restricted to US reports and to reports received by 25 April 2024 (the most recent record in the publicly released 2024 file at the time of analysis); later reports referring to vaccinations administered before April 2024 will appear in future VAERS releases. Fifth, the Shingrix two-dose schedule was not modeled at the dose level, because the VAERS data file does not consistently capture dose number; reactogenicity is known to be more pronounced after dose 2 and the present aggregate estimates therefore mix dose-1 and dose-2 reports. Sixth, reports in which another vaccine was co-administered (5,199 reports; 7.4%) were retained because co-administration reflects routine clinical practice; reported reactogenicity in these reports cannot be attributed to the herpes zoster vaccine alone.

Three further limitations relate to the structure of the publicly released VAERS data set. Seventh, when more than one report describes the same case, only the first report received is retained in the publicly accessible dataset; subsequent reports may contain additional or corrected information that is not visible to external analysts (19). Eighth, although VAERS staff routinely follow up on serious and selected non-serious reports to obtain medical, laboratory, or autopsy records, the MedDRA preferred terms originally coded for a report are not generally updated on the basis of follow-up information; the public file therefore reflects the initial coding rather than the most current clinical understanding of each case (19). Ninth, MedDRA is updated semi-annually, and the same clinical event may be coded with slightly different preferred terms across the 8 years of the present study (approximately 16 successive MedDRA versions). We grouped clinically related preferred terms into MedDRA system organ classes for the disproportionality analysis to mitigate this drift, but residual misclassification at the preferred-term level is possible (19).

### 4.5 Implications

The findings reinforce existing public-health messaging about Shingrix: clinicians should counsel recipients—particularly those receiving dose 2—to expect transient constitutional symptoms (most commonly fever, chills, fatigue, headache, and myalgia) that typically begin within 24 h of vaccination and resolve within a few days. Anticipatory guidance about reactogenicity may support completion of the two-dose series and reduce unscheduled healthcare utilization. For Zostavax, which is no longer in US use, the disproportionate reporting of herpes zoster and vesicular rash following the live vaccine underscores the rationale for the ACIP-preferred status of the recombinant Shingrix vaccine, especially in older adults and immunocompromised populations (16, 17). The age-related increase in the proportion of serious reports is consistent with the heightened baseline vulnerability of older adults rather than with a vaccine-specific safety signal and should not deter vaccination, given the substantially greater incidence and severity of natural shingles in this population.

### 4.6 Future research

Several areas warrant further work. First, dose-stratified active-surveillance studies would clarify the true incidence of reactogenic events after Shingrix dose 1 versus dose 2 in real-world populations. Second, integration of VAERS signals with the Vaccine Safety Datalink and Medicare claims databases would permit risk-difference estimation for outcomes such as Guillain-Barré syndrome, immune thrombocytopenia, and acute cardiovascular events, building on existing post-licensure work (28). Third, evaluation of Shingrix safety in immunocompromised adults aged 19 years and older—newly indicated since 2021—would address an evidence gap not captured by the pre-licensure trials in immunocompetent older adults.

## 5 Conclusion

In this descriptive analysis of 69,822 US VAERS reports (2017–2024), reporting for both herpes zoster vaccines was dominated by transient, non-serious reactogenicity, and serious reports were uncommon (6.6% overall; 2.9% for the recombinant vaccine, Shingrix). Reports of herpes zoster and vesicular rash following vaccination (not virologically confirmed as vaccine-strain, and subject to protopathic bias) were concentrated among the live vaccine, Zostavax, consistent with its attenuated-virus biology and now largely of historical relevance given the US transition to the non-live recombinant vaccine. The practical message for clinicians is reassuring: when administering Shingrix, counsel patients to expect short-lived local and systemic reactions in the first few days, advise simple symptomatic care, and emphasize that serious events are rare; suspected adverse events should continue to be reported to VAERS. Because passive-surveillance data lack denominators and cannot establish causality, these findings describe reporting patterns rather than risk and should be confirmed by active-surveillance studies with appropriate comparators.

## Data Availability

Publicly available datasets were analyzed in this study. This data can be found here: https://vaers.hhs.gov/data.html.
