# Supplementary material for: Safety profile of herpes zoster vaccines in the United States: a descriptive and disproportionality analysis of adverse events reported to the vaccine adverse event reporting system (VAERS), 2017–2024
Source: Front Med (Lausanne). 2026 Aug 13;13:1920596. doi: 10.3389/fmed.2026.1920596 (PMC13518110; doi:10.3389/fmed.2026.1920596)
Supplement: Supplementary file 1 [file Table_1.docx]

Supplementary Material

# Supplementary Tables

**Supplementary Table S1.** Distribution of U.S. herpes zoster vaccine adverse-event reports by year of receipt and vaccine brand, VAERS 2017–2024.

| **Year of receipt** | **Total n (% of cohort)** | **Shingrix n (% of year)** | **Zostavax n (% of year)** | **Unspecified n (% of year)** |
| --- | --- | --- | --- | --- |
| 2017 | 3,517 (5.0%) | 8 (0.2%) | 3,486 (99.1%) | 23 (0.7%) |
| 2018 | 16,785 (24.0%) | 13,285 (79.1%) | 3,455 (20.6%) | 45 (0.3%) |
| 2019 | 16,774 (24.0%) | 15,079 (89.9%) | 1,656 (9.9%) | 39 (0.2%) |
| 2020 | 11,078 (15.9%) | 9,887 (89.2%) | 1,107 (10.0%) | 84 (0.8%) |
| 2021 | 8,833 (12.7%) | 8,089 (91.6%) | 609 (6.9%) | 135 (1.5%) |
| 2022 | 5,751 (8.2%) | 5,560 (96.7%) | 141 (2.5%) | 50 (0.9%) |
| 2023 | 5,537 (7.9%) | 5,378 (97.1%) | 48 (0.9%) | 111 (2.0%) |
| 2024† | 1,547 (2.2%) | 1,527 (98.7%) | 5 (0.3%) | 15 (1.0%) |
| Total | 69,822 (100.0%) | 58,813 (84.2%) | 10,507 (15.0%) | 502 (0.7%) |

*Notes: Percentages in the “Total” column are calculated against the overall cohort (n = 69,822). Percentages in the brand columns are calculated against the within-year total. Unspecified denotes reports in which the vaccine brand could not be determined from the VAERS record. † Through April 2024 only (partial year).*

**Supplementary Table S2.** Stratified counts of U.S. herpes zoster vaccine VAERS reports and the proportion meeting U.S. FDA serious-adverse-event criteria, by age group, sex, and place of vaccine administration, 2017–2024.

| **Stratum** | **Reports n (% of cohort)** | **Serious reports n (% of stratum)** |
| --- | --- | --- |
| Age group, n (% of cohort) |  |  |
| 18–49 | 670 (1.0%) | 13 (1.9%) |
| 50–59 | 15,084 (21.6%) | 410 (2.7%) |
| 60–69 | 20,778 (29.8%) | 702 (3.4%) |
| 70–79 | 11,865 (17.0%) | 497 (4.2%) |
| ≥80 | 3,239 (4.6%) | 166 (5.1%) |
| Unknown | 18,186 (26.0%) | 2,809 (15.4%) |
| Sex, n (% of cohort) |  |  |
| Female | 44,605 (63.9%) | 2,280 (5.1%) |
| Male | 19,169 (27.5%) | 1,448 (7.6%) |
| Unknown / undifferentiated | 6,048 (8.7%) | 869 (14.4%) |
| Place of administration, n (% of cohort) |  |  |
| Pharmacy | 33,194 (47.5%) | 1,757 (5.3%) |
| Private/physician's office | 8,784 (12.6%) | 612 (7.0%) |
| Public health clinic | 1,335 (1.9%) | 54 (4.0%) |
| Other facility | 4,923 (7.1%) | 484 (9.8%) |
| Other / Unknown | 21,586 (30.9%) | 1,690 (7.8%) |

*Notes: Percentages in the “Reports” column are calculated against the overall cohort (n = 69,822). Percentages in the “Serious reports” column are calculated against the within-stratum total. A serious report is one that meets at least one of the U.S. FDA criteria (death, life-threatening event, hospitalization, prolongation of hospitalization, or permanent disability) as captured by the VAERS report form. Sub-stratum header rows (in italics) display the stratifier label and contain no counts.*

**Supplementary Table S3.** Brand-stratified reporting odds ratios (RORs) for reactogenicity and systemic MedDRA preferred terms, U.S. herpes zoster vaccine VAERS reports, 2017–2024. Each brand and the combined cohort are compared against the same external comparator (all other U.S. VAERS reports, n = 1,029,498); the brands are not compared with each other.

| **Preferred term** | **Shingrix ROR (95% CI)** | **Zostavax ROR (95% CI)** | **Combined ROR (95% CI)** |
| --- | --- | --- | --- |
| Influenza-like illness | 5.37 (5.17–5.59) | 0.28 (0.20–0.39) | 4.55 (4.37–4.73) |
| Injection-site pain | 3.42 (3.35–3.51) | 0.58 (0.52–0.64) | 2.92 (2.86–2.99) |
| Injection-site erythema | 3.05 (2.97–3.13) | 1.47 (1.36–1.59) | 2.78 (2.71–2.85) |
| Injection-site swelling | 2.53 (2.46–2.61) | 1.20 (1.09–1.32) | 2.31 (2.24–2.38) |
| Myalgia | 2.34 (2.28–2.41) | 0.14 (0.11–0.18) | 1.98 (1.92–2.03) |
| Erythema | 2.27 (2.20–2.35) | 0.93 (0.83–1.03) | 2.06 (1.99–2.12) |
| Chills | 2.18 (2.14–2.23) | 0.09 (0.07–0.11) | 1.81 (1.77–1.84) |
| Pain in extremity | 1.91 (1.86–1.95) | 0.20 (0.17–0.23) | 1.61 (1.57–1.65) |
| Pyrexia | 1.89 (1.86–1.93) | 0.13 (0.11–0.15) | 1.57 (1.54–1.60) |
| Pain | 1.88 (1.84–1.93) | 1.23 (1.17–1.31) | 1.78 (1.74–1.82) |
| Rash | 1.88 (1.83–1.94) | 1.88 (1.76–2.02) | 1.88 (1.83–1.94) |
| Malaise | 1.77 (1.70–1.84) | 0.19 (0.15–0.25) | 1.52 (1.46–1.58) |
| Headache | 1.40 (1.37–1.43) | 0.15 (0.13–0.17) | 1.18 (1.15–1.20) |
| Nausea | 1.36 (1.32–1.40) | 0.12 (0.10–0.15) | 1.15 (1.12–1.18) |
| Fatigue | 1.35 (1.32–1.39) | 0.08 (0.07–0.10) | 1.13 (1.11–1.16) |
| Asthenia | 1.28 (1.23–1.33) | 0.20 (0.16–0.25) | 1.11 (1.07–1.15) |
| Arthralgia | 1.21 (1.17–1.26) | 0.17 (0.14–0.21) | 1.05 (1.01–1.08) |
| Pruritus | 1.21 (1.17–1.26) | 0.61 (0.54–0.69) | 1.12 (1.08–1.17) |
| Hypersensitivity | 1.19 (1.07–1.32) | 0.63 (0.46–0.88) | 1.12 (1.02–1.24) |
| Back pain | 1.16 (1.09–1.23) | 0.33 (0.25–0.43) | 1.03 (0.97–1.09) |
| Urticaria | 0.95 (0.91–1.00) | 0.28 (0.23–0.35) | 0.85 (0.81–0.89) |
| Diarrhoea | 0.92 (0.87–0.97) | 0.09 (0.06–0.13) | 0.79 (0.75–0.83) |
| Paraesthesia | 0.76 (0.72–0.81) | 0.32 (0.26–0.40) | 0.69 (0.65–0.73) |
| Vomiting | 0.73 (0.69–0.76) | 0.11 (0.08–0.14) | 0.63 (0.60–0.66) |
| Dizziness | 0.63 (0.61–0.65) | 0.12 (0.10–0.15) | 0.55 (0.53–0.57) |
| Anaphylactic reaction | 0.42 (0.32–0.55) | 0.35 (0.17–0.70) | 0.41 (0.32–0.53) |

*ROR, reporting odds ratio; CI, confidence interval. Each ROR compares reporting of the event in the indicated exposure group with reporting in all other U.S. VAERS reports (the same external comparator for every column); this is a signal-detection screen and NOT a between-brand comparison, and no between-brand statistical test was performed. RORs are unadjusted and do not estimate risk. Zostavax cell counts are small for several terms, yielding wider confidence intervals; the two brands were also reported in largely non-overlapping calendar periods against a comparator whose composition changed over time (temporal confounding). Disease-overlap preferred terms (herpes zoster, vesicular rash, vaccination failure) are deliberately omitted because a brand-stratified ROR for these terms would be dominated by confounding by indication / protopathic bias (reported vaccine-strain or breakthrough disease), the same rationale for which no herpes zoster ROR is reported in the main text.*

**Supplementary Table S4.** Full breakdown of place of vaccine administration with serious-report proportions, U.S. herpes zoster vaccine VAERS cohort, 2017–2024 (N = 69,822).

| **Place of administration (VAERS code)** | **Reports, n (% of cohort)** | **Serious reports, n (%)** |
| --- | --- | --- |
| Pharmacy | 33,194 (47.5%) | 1,757 (5.3%) |
| Not specified / unknown | 20,840 (29.8%) | 1,621 (7.8%) |
| Private or physician's office | 8,784 (12.6%) | 612 (7.0%) |
| Other facility | 4,923 (7.1%) | 484 (9.8%) |
| Public health clinic | 1,335 (1.9%) | 54 (4.0%) |
| Military | 417 (0.6%) | 56 (13.4%) |
| Workplace | 238 (0.3%) | 4 (1.7%) |
| Senior-living facility | 54 (0.1%) | 7 (13.0%) |
| School | 37 (0.1%) | 2 (5.4%) |

*Place of administration is a single coded VAERS field (V_ADMINBY) with one value per report; the “Other facility” (OTH) code is an atomic category that the data dictionary illustrates with examples (long-term care, occupational, hospital outpatient) but that cannot be decomposed further from the structured data. Several granular codes (pharmacy, school, senior-living, workplace) are available only on the VAERS-2 form; legacy VAERS-1 reports (n = 3,516; 5.0%) cannot be classified into those settings. Serious = any FDA serious-adverse-event criterion. Between-setting differences should be interpreted as case-mix (correlated with brand, calendar period, and recipient characteristics), not vaccine risk.*

**Supplementary Table S5.** Missing-age sensitivity analysis: serious-report proportion among reports with documented versus missing recipient age, overall and by brand, U.S. herpes zoster vaccine VAERS cohort, 2017–2024.

| **Group** | **Documented age, n** | **Serious % (documented age)** | **Missing age, n** | **Serious % (missing age)** | **% of reports with age missing** |
| --- | --- | --- | --- | --- | --- |
| Overall cohort | 51,636 | 3.5% | 18,186 | 15.4% | 26.0% |
| Shingrix | 48,397 | 3.2% | 10,416 | 1.5% | 17.7% |
| Zostavax | 3,043 | 8.2% | 7,464 | 35.5% | 71.0% |

*Age missingness is strongly differential (71.0% for Zostavax versus 17.7% for Shingrix) and is associated with the earlier reporting era and the legacy VAERS-1 form, indicating a not-missing-at-random pattern; age was therefore not imputed. Because missing-age reports are disproportionately Zostavax (which have a higher serious-report proportion), the overall serious proportion appears higher among missing-age reports, whereas within Shingrix the pattern reverses—illustrating that a single imputation model could not capture the mechanism. The main-text age-stratified analysis is a complete-case analysis; the monotonic increase in serious-report proportion with age (1.9% at 18–49 years to 5.1% at ≥80 years) persists among reports with documented age and is most representative of the Shingrix, VAERS-2-era cohort.*

# Supplementary Figures

**Supplementary Figure S1.** Study flow diagram. Of 85,422 reports listing a herpes zoster vaccine (VAERS vaccine type VARZOS) received 2017–2024, 15,600 without a recorded U.S. state, the District of Columbia, or a U.S. territory were excluded, yielding the analytic cohort of 69,822 reports (Shingrix 58,813 [84.2%]; Zostavax 10,507 [15.0%]; unspecified brand 502 [0.7%]). No reports were excluded for missing demographic or clinical fields. Brand was assigned from the vaccine-name field (10 reports listing both brands were assigned to Shingrix).
